# Linoleic Acid: A Nutritional Quandary

**DOI:** 10.3390/healthcare5020025

**Published:** 2017-05-20

**Authors:** Ronald J. Jandacek

**Affiliations:** Department of Pathology and Laboratory Medicine, University of Cincinnati, Cincinnati, OH 45237, USA; Ronald.jandacek@uc.edu; Tel.: +1-513-558-5492

**Keywords:** linoleic acid, atherosclerosis, dietary recommendations

## Abstract

Over the course of the twentieth century, there was a 20-fold increase in consumption of vegetable oils resulting both from their increased availability and from recommendations to consume these oils as an aid to lower blood cholesterol levels. This dietary change markedly increased the consumption of linoleic acid to current levels of approximately 6% of total dietary energy. While considerable research has focused on the effects of dietary linoleic acid on cardiovascular health, questions about optimum dietary levels remain. For example, meta-analyses disagree about the role of dietary linoleic acid in atherosclerosis, and recent publications indicate that linoleic acid’s reduction of blood cholesterol levels does not predict its effect on the development of atherosclerosis. Further, there are also detrimental effects of elevated dietary linoleic acid on human health related to its role in inflammation and its activity as a promoter of cancer in animals. Current data do not allow determination of the level of dietary linoleic acid needed for optimum health. Studies of the effects of a wide range of linoleic acid consumption may help determine dietary recommendations that are optimal for human health.

## 1. Introduction

Understanding the nutritional value of linoleic acid is important from many viewpoints. The purpose of this review and analysis is to focus on what is known, what is unknown, and what is contentious about dietary linoleic acid and health, as well as to recommend areas for further study. Several points are important.

First, there was an enormous discontinuity in the intake of linoleic acid during the last century as the availability of vegetable oils containing linoleic acid increased 20-fold, markedly increasing average linoleic acid consumption in the U.S. [1]. This change was the result of increased commercial availability combined with dietary recommendations emphasizing the presumed health benefits of linoleic acid; and because of its action to reduce blood cholesterol, there were essentially no prospective data related to the impact of this unprecedented dietary change on areas other than the development of atherosclerosis.

Second, the generally accepted benefit of linoleic acid in slowing the development of atherosclerosis has come into question. Specifically, three large meta-analyses carried out by well-established research groups have produced conflicting results, making it extremely difficult for nutrition scientists to determine optimal dietary intake levels that are appropriate for health [2,3,4].

Third, in the body linoleic acid elongates and desaturates to form arachidonic acid, a precursor to pro-inflammatory compounds that can have detrimental effects on health. Understanding the importance of these “side effects” of consuming linoleic acid has led to the widely adopted approach of countering these n-6 pro-inflammatory fatty acids with n-3 fatty acids, including α-linolenic acid from vegetable sources and eicosapentaenoic and docosahexaenoic acids (EPA and DHA, respectively) found in some fish oils. This common practice begs the question as to whether alternative approaches might be possible to counter the pro-inflammatory effect or whether in fact consuming so much linoleic acid is appropriate and healthful.

Fourth, a relationship between dietary linoleic acid and the development of cancer has been suggested in numerous reports (see e.g., [5,6]). While the data cannot be said to be conclusive, they nevertheless support the recommendation that further study is needed to address a potential causative role of linoleic acid in cancer.

Fifth, novel cultivars of vegetable oils have recently appeared with markedly lower levels of linoleic acid than the traditional cultivars. For the most part, these new oil seeds contain oleic acid in place of linoleic acid. As these new seeds become widely available, they could potentially create a large shift in the U.S. diet back to linoleic acid levels comparable to those at the beginning of the twentieth century. It is consequently highly important to understand the health effects of this second dramatic shift in nutrition before it happens rather than after it happens as occurred for the large increase in linoleic acid intake.

Sixth, although one must consider the data anecdotal, studies of societies with optimum health and longevity may provide some insight into the need for linoleic acid in the diet [7]. These reports are qualitative but potentially instructive.

Lastly, given the complexity of the nutritional role of linoleic acid, all of these areas should be considered in determination of an appropriate level of intake. The history of dietary recommendations for linoleic acid consists of a transition from early quantitative values into more subjective recommendations without numerical units.

## 2. The Change in Linoleic Acid Intake

At the beginning of the 1900s, fats from animal sources dominated the diet in the U.S. Butter, lard, and tallow were used for spreads, baking, and frying. Although these fats contained only small quantities of linoleic acid, the level was presumably sufficient to prevent essential fatty acid deficiency, which was characterized by skin and hair disorders and later quantitatively defined by a ratio of eicosatrienoic acid to arachidonic acid of greater than 0.4 [8].

Two driving forces produced a major change in fat consumption. First, vegetable oil shortening and oils were developed and marketed as having apparent functionality equivalence with animal fats and with substantial advantages for cost.

The availability of vegetable oils, which essentially is the amount sold including unconsumed waste, in 1909 was 0.7 kg/person/year [1]. The comparable figure in 1999 was 14.7 kg/person/year, a 20-fold increase. Based on the U.S. Department of Agriculture reports, current consumption of linoleic acid by average adult men in the U.S. is 16.0 g per day, and by adult women, 12.6 g/day [9]. These intake levels correspond to 6.0% and 5.5% of total average consumed energy, respectively [10].

The second driving force underlying the dietary shift was related to health benefits. Although the increase took place with essentially no understanding of its effects on short-term or long-term health, the influential studies of Keys and others discussed below led many to conclude that dietary linoleic acid would reduce blood cholesterol levels and thereby lower the risk of the development of atherosclerosis. Organizations such as the American Heart Association recommended increases in consumption of polyunsaturated fat, which equated with increases in linoleic acid consumption.

## 3. Recent Assessments of Linoleic Acid and the Risk of Atherosclerosis

In the mid-1950s, Ancel Keys and his colleagues reported two kinds of studies that placed dietary linoleic acid in a favorable light with regard to the development of atherosclerosis and coronary artery disease. He analyzed diets and health in seven countries and found that atherosclerosis and mortality were positively correlated with a high intake of saturated fatty acids [11]. The incidence of heart disease differed considerably among countries and was highest in those with the highest average intake of saturated fatty acids. Keys also reported the results of dietary experiments that were carried out on men who received well defined and characterized menus [12]. He observed a relationship between the blood cholesterol levels of the subjects and the type of fatty acids that they ingested, and this relationship became known as the Keys Equation. The equation states that a change in blood cholesterol (mg/dL) is equal to 2.7 times the change in saturated fatty acid intake minus 1.3 times the change in polyunsaturated fatty acid intake. The units of the fatty acids in this equation were percent of energy intake, so that a 10% increase in energy consumed as linoleic acid would correspond to a 13 mg/dL decrease in blood cholesterol. The measurements were in terms of total blood cholesterol, since LDL and HDL cholesterol were not yet routinely measured.

These studies were highly influential in terms of available food products and dietary recommendations from authoritative health organizations. Although it is not possible to quantify the effect precisely, there was a significant impact on the U.S. diet. Specifically, the ready supply and extensive marketing of vegetable oil and vegetable oil products resulted in the displacement of butter, shortening, and tallow frying fats. These linoleic acid-based oils resulted in an increase in the intake of polyunsaturated fatty acids and a partial replacement of saturated fatty acid-based products, consistent with the Keys equation formula for reducing blood cholesterol levels.

Recent studies of the effects of dietary linoleic acid on the risk of atherosclerosis present conflicting results. Specifically, three large meta-analyses have addressed the relationship between the amount of linoleic acid in the diet and incidence of cardiovascular disease. 

Farvid et al. reported the analysis of cohort studies with 310,602 subjects and reached the conclusion that dietary linoleic acid in the highest category of linoleic acid intake corresponded to a 15% lower risk of coronary heart disease relative to the lowest category [2]. The replacement of 5% of energy as saturated fatty acids by linoleic acid was associated with a 9% reduction in coronary disease.

Different results and conclusions were reported by Chowdhury and coworkers [3]. Reviewing studies of 530,525 participants, they observed no benefit of linoleic acid supplementation with regard to coronary disease. They also found that saturated fatty acid intake did not increase risk. They concluded that their data do not support guidelines to reduce saturated fat intake and/or increase polyunsaturated fat intake to reduce the risk of coronary disease.

In 2016 Ramsden and colleagues reviewed studies that raised further doubts about the benefits of linoleic acid with regard to the reducing coronary disease. First, they analyzed data from a double-blind diet study of 9423 women and men carried out in 1968–1973 [4]. This study examined institutionalized subjects who consumed a dietary regimen similar to that used by Keys, and additionally included post-study assessment of the health of the subjects. They found that linoleic acid reduced blood cholesterol levels by 13.8% in the manner predicted by the Keys equation. However, they more surprisingly found that there was a 22% increase in the risk of death for each 30 mg/dL reduction in serum cholesterol. The investigators also performed a meta-analysis of 10,808 subjects in five randomized controlled studies and found no beneficial effect of dietary linoleic acid on the development of cardiovascular disease. They further corroborated these findings with data from a retrospective study of 458 men in Sydney, Australia in which dietary linoleic acid did not reduce the development of heart disease [13]. It should be emphasized that these studies of institutionalized subjects in 1968–1973 and also in Sydney were intervention trials with well-defined dietary intake thereby eliminating concerns about errors in dietary intake assessment associated with epidemiological trials.

Another approach to considering the possible benefit of linoleic acid in cardiovascular health was made by Wu et al. [14]. Levels of linoleic acid in plasma phospholipids in 2792 participants were used as surrogate indicators of linoleic acid consumption, and higher levels of linoleic acid were associated with lower total mortality that was attributed to lower incidence of cardiovascular disease.

These studies illustrate the difficulty in assessing the nutritional effects of linoleic acid. A definitive conclusion as to whether linoleic acid is beneficial to cardiovascular health cannot be made since the current state of publication leaves one with little guidance.

## 4. Inflammation

Arachidonic acid, a 20-carbon fatty acid, is synthesized from linoleic acid. It is the precursor to prostaglandins and other eicosanoids that are inflammatory and that enhance platelet aggregation [15]. Both linoleic and arachidonic are n-6 fatty acids, and considerable research has been directed to the use of n-3 fatty acids as a means of reducing the inflammatory effects of the n-6 acids. The rationale is based on the production of classes of compounds that are less inflammatory than those produced from the n-6 acids and competition for enzymes that convert arachidonic acid to inflammatory substances. The often used approach to ameliorate inflammation due to linoleic acid has been to reduce the ratio of n-6 to n-3 acids in the diet by increasing dietary n-3 acids. Based on the belief that increased consumption of linoleic acid is beneficial to the cardiovascular system, and the recognition that some of its n-6 metabolites are pro-inflammatory, diets already enhanced with linoleic acid are often fortified with n-3 fatty acids as well.

An alternative approach, but one that has generally been overlooked, is that of reducing the inflammation resulting from linoleic and arachidonic acids by simply reducing the level of these acids in the diet without the need to increase n-3 consumption. Decreasing the n-6 to n-3 ratio in the diet and consequently in the organism can be attained not only by increasing dietary n-3 acids but also by decreasing dietary n-6 acids. Although the exact requirement for the level of linoleic acid to prevent fatty acid deficiency is not known, based on other mammalian requirements a reasonable estimate is 1–2% of energy [8]. Since consumption by people in the U.S. is more than 3 times that level, it should be possible to test the levels of linoleic acid that are markedly less than current consumption while meeting the essential fatty acid requirement.

A recent review of linoleic acid’s role in inflammation and the pathogenesis of obesity discusses this approach of lowering linoleic acid intake to reduce inflammatory effects [16]. In addition, Liou et al. reported that reducing linoleic acid intake in men increased the level of n-3 eicosapentaenoic acid in plasma phospholipids in men [17]. Rodrigues et al. recently reported that hastening of inflammation with dietary linoleic acid in a diabetic rat improved wound healing in a diabetic rat [18] 

## 5. Cancer

There is no direct evidence that linoleic acid is a carcinogen. There is, however, compelling evidence that in some species and under some conditions linoleic acid can act as a promoter of tumor growth. In one report the rate of growth of tumors in animals challenged with a carcinogen linearly increased with increasing levels of dietary linoleic acid until a threshold was reached when linoleic acid accounted for 10% of energy [19]. Similar results have been reported from other studies, for example, linoleic acid-based oils have been found to promote mammary cancer in rats [20]. Studies of colon cancer are inconclusive in that there are reports that linoleic acid may be either beneficial or associated with enhanced severity of colon cancer in carcinogen-challenged rats [21,22]. Sauer and coworkers reviewed the effects of dietary factors on experimental tumors and found that linoleic acid “is responsible for up-regulation of tumor growth in vivo” ([5] p. 637).

Data from human studies generally do not implicate linoleic acid in the development of cancer. However, one clinical experiment that was designed to test the hypothesis that increased levels of dietary linoleic acid would reduce the risk of heart disease actually found an increase in the incidence of cancer and cancer mortality in subjects assigned to the high-linoleic acid diet [23]. A review and meta-analysis of animal studies, prospective, case control, and ecological human trials by Zock and Katan found no relation between colorectal, breast or prostate cancer with dietary linoleic acid, although the authors concluded that the data cannot exclude a small increase in risk [6]. Azrad and coworkers reviewed the relationship of polyunsaturated fatty acids to cancer risk and concluded that “epidemiologic studies provide an inconsistent picture of the associations between dietary PUFAs and cancer”, including both n-3 and n-6 PUFAs (polyunsaturated fatty acids) ([24] p. 1). Bartsch and coworkers reviewed human and animal data and concluded that linoleic acid is implicated in cancer of the breast and colon, and possibly the prostate [25].

There is a rationale for implicating linoleic acid in tumor growth. If like other cells tumor cells require linoleic/arachidonic acid for the synthesis of functional cell membranes, then one might reason that depriving the membrane of the source of these acids with a diet containing minimal levels of linoleic acid might slow tumor growth by reducing the substrate required for membrane formation. The use of a very low linoleic acid diet could be tested in combination with standard anti-cancer therapies with minimal risk. It is certainly possible that different cancers may respond differentially to linoleic acid availability.

A recent meta-analysis of breast cancer risk in 358,955 women in several countries found a benefit of dietary linoleic acid [26]. The risk of breast cancer decreased by 1% for each 10 g/day increment in linoleic acid ingestion.

There are scant reports of tumor membrane fatty acid composition. Ovarian and endometrial cancer cells utilize essential fatty acids [27]. Although there have been some other studies of tumor fatty acids, in general little is known about the requirement for essential fatty acids in different cancers [28,29].

There are correlative data that support the idea that increases in the incidence of some cancers mirror the increase in linoleic acid consumption. Hallberg and Johansson reported marked increases in prostate cancer, breast cancer, melanoma, lung cancer, and bladder cancer beginning in the middle of the twentieth century, reflecting the same period in which linoleic acid intake increased [30]. Certainly other factors such as smoking, background radiation, and other environmental changes may also show similar correlations, but the association with linoleic acid should not be ignored.

## 6. New Cultivars

A novel discontinuity in edible oil nutrition appears to be in progress. The removal of *trans* fatty acids from many foods has indirectly resulted in the development of oil seed cultivars that will undoubtedly impact current consumption of linoleic acid [31]. One of the functions of the hydrogenation of edible oils was a reduction in the number of double bonds that are susceptible to oxidation in frying applications. Because a byproduct of hydrogenation is the production of *trans* acids, the elimination of hydrogenation has led to alternative approaches to making oxidatively stable fats. This effort has resulted in the development of cultivars that are lower in the degree of unsaturation. Hence, linoleic acid has been replaced with the more stable oleic acid. High oleic acid sunflower oil and safflower oil, oils that previously were high in linoleic acid, are now readily available to consumers. More importantly in terms of cost and availability, high oleic acid soybeans are now available.

The commercial availability and desirability of high oleic (low linoleic) acid vegetable oils will presumably reduce the consumption of linoleic acid. It is not unreasonable to predict that linoleic acid in the diet may revert to its level of a century ago. This major shift in diet composition would appear to be a safe transition without detrimental effects on health. In some cultures humans have been ingesting diets high in oleic acid in the form of olive oil for millennia, much longer than the century of human experience with high linoleic acid oil consumption. The healthfulness of the “Mediterranean diet” is often associated with olive oil consumption. It also should be noted that unlike linoleic acid, oleic acid is synthesized de novo in the body [32]. Body stores of oleic acid arise both from endogenous and exogenous sources, so that human metabolism of oleic acid has evolved for millennia.

Related to the potential increase in dietary oleic acid, in terms of effects on lipoprotein cholesterol markers that are generally associated with the risk of atherosclerosis development, there is evidence that consumption of high oleic acid oils results in favorable levels of LDL cholesterol [31]. There are also indications that high oleic acid oils may maintain HDL cholesterol levels higher than high linoleic acid oil [33].

## 7. Anecdotal Evidence

Studies of geographic regions characterized by longevity accompanied by low levels of heart disease, obesity, cancer, and diabetes may be instructive in the understanding the need for linoleic acid in the human diet [7]. Buettner studied five areas that met the criteria of longevity and long-term health: Ikaria, Greece; Okinawa, Japan; Ogliastra Region, Sardinia; Loma Linda, California; and Nicoya Peninsula, Costa Rica.

Based on published estimates of dietary intakes from these regions, in all cases the consumption of linoleic acid in these areas appears to be below current intakes in the U.S. For example, in Ikaria, the only fat added to the diet was olive oil, which accounted for 6% of the mass of daily intake. This level would correspond to approximately 12% of energy as olive oil, and +thereby less than 2% of energy as linoleic acid.

As noted, this kind of evidence is circumstantial, but it does seem to illustrate that in some cultures it is possible to live an extended healthy life with very low consumption of linoleic acid. It is not clear if this conclusion can be extrapolated to groups of people with diverse genetic backgrounds, energy expenditure, and energy consumption.

## 8. Conclusions: Dietary Recommendations

In the wave of the work of Keys and others, health organizations including the American Heart Association, recommended high levels of linoleic acid intake. This recommendation pattern continued through the 1970s with a recommendation that total fat consumption be less than 30% of energy and that polyunsaturated fat account for 10 of the 30%. As knowledge about the pro-inflammatory properties of linoleic acid emerged, emphasis shifted to reducing saturated fatty acid intake, and recommendations for linoleic acid began to be downplayed. As discussed below, current assessments of linoleic acid have essentially concluded that the current level of linoleic acid consumption is appropriate.

In 2014, the Academy of Nutrition and Dietetics reported its position on dietary fatty acid consumption [34]. The report emphasized the inclusion of n-3 fatty acids and also summarized recommendations for linoleic acid intake from organizations including the Dietary Guidelines for America, American Heart Association, and the WHO. These recommendations include “using oils to replace solid fats”, including linoleic acid at a level of 2% of energy to meet essential fatty acid needs, and linoleic acid up to a level of 10% of energy, presumably to provide cardiovascular benefits. Given the quandary about the putative benefit of linoleic acid in heart disease, and the increasing availability of high oleic acid oils, it seems reasonable to consider a level that is equal to that required to prevent essential fatty acid deficiency symptoms. This level is generally thought to be 1–2% of energy [8]. The work of Holman with infants determined that 1.4% of energy as linoleic acid was sufficient [35].

In spite of the numerous studies of linoleic acid metabolism, current data are insufficient to allow the determination of a recommended universal intake above that required to meet essential fatty acid requirements. It seems unlikely that future studies will unequivocally determine its effects on the development of atherosclerosis. A complete understanding is confounded by the various ways that linoleic acid can be included in the diet whether as a substitution for saturated, trans, or n-3 fats [36,37]. In addition, there seems to be no clear path to understanding if it has a role in the promotion of cancer, although it is intriguing to consider the use of very low linoleic acid diets to hinder tumor growth by deprivation of cell membrane structural requirements.
